# Postcranial disparity of galeaspids and the evolution of swimming speeds in stem-gnathostomes

**DOI:** 10.1093/nsr/nwad050

**Published:** 2023-02-27

**Authors:** Zhikun Gai, Xianghong Lin, Xianren Shan, Humberto G Ferrón, Philip C J Donoghue

**Affiliations:** Key Laboratory of Vertebrate Evolution and Human Origins of Chinese Academy of Sciences, Institute of Vertebrate Paleontology and Paleoanthropology, Chinese Academy of Sciences, Beijing 100044, China; University of Chinese Academy of Sciences, Beijing 100049, China; Key Laboratory of Vertebrate Evolution and Human Origins of Chinese Academy of Sciences, Institute of Vertebrate Paleontology and Paleoanthropology, Chinese Academy of Sciences, Beijing 100044, China; Key Laboratory of Vertebrate Evolution and Human Origins of Chinese Academy of Sciences, Institute of Vertebrate Paleontology and Paleoanthropology, Chinese Academy of Sciences, Beijing 100044, China; University of Chinese Academy of Sciences, Beijing 100049, China; Bristol Palaeobiology Group, School of Earth Sciences, University of Bristol, BristolBS8 1TH, UK; Instituto Cavanilles de Biodiversidad i Biología Evolutiva, Universitat de València, Paterna 46980, Valencia, Spain; Bristol Palaeobiology Group, School of Earth Sciences, University of Bristol, BristolBS8 1TH, UK

**Keywords:** Galeaspida, jawed vertebrates, evolution, functional morphology, phylogenetics, modelling

## Abstract

Galeaspids are extinct jawless relatives of living jawed vertebrates whose contribution to understanding the evolutionary assembly of the gnathostome bodyplan has been limited by absence of postcranial remains. Here, we describe *Foxaspis novemura* gen. et sp. nov., based on complete articulated remains from a newly discovered Konservat-Lagerstätte in the Early Devonian (Pragian, ∼410 Ma) of Guangxi, South China. *F. novemura* had a broad, circular dorso-ventrally compressed headshield, slender trunk and strongly asymmetrical hypochordal tail fin comprised of nine ray-like scale-covered digitations. This tail morphology contrasts with the symmetrical hypochordal tail fin of *Tujiaaspis vividus*, evidencing disparity in galeaspid postcranial anatomy. Analysis of swimming speed reveals galeaspids as moderately fast swimmers, capable of achieving greater cruising swimming speeds than their more derived jawless and jawed relatives. Our analyses reject the hypothesis of a driven trend towards increasingly active food acquisition which has been invoked to characterize early vertebrate evolution.

## Teaser

Zhikun Gai and colleagues describe the extinct jawless fish Foxaspis novemura, only the second galeaspid known from complete remains. Their analysis of swimming speeds based on tail morphology reveals that relatives of the ancestral jawed vertebrate were ecologically diverse, rejecting the hypothesis of a trend to increasingly active food acquisition.

## INTRODUCTION

Almost all living vertebrates are jawed vertebrates but they evolved from jawless ancestors, the living relatives of which are the cyclostomes (lampreys and hagfishes) that are significantly reduced relative to the bodyplan of the ancestral vertebrate [1]. Consequently, attempts to explain the evolutionary assembly of the gnathostome bodyplan are complicated by the primitive and secondary absence of characters in cyclostomes, which are difficult to discriminate. Nevertheless, there is a rich fossil record of jawless and jawed vertebrates related by degree to the living jawed vertebrates, recording the sequential assembly of the gnathostome bodyplan [2,3]. These ‘ostracoderms’ are characterized by their extensively developed dermal boney armour [4], preserving aspects of external and internal anatomy, from gross aspects of anatomy to fine details of cranial innervation [5]. These data have been influential in testing and informing hypotheses that seek to explain the origin of jawed vertebrates in terms of developmental evolution [6–9].

Most of the component lineages of stem-gnathostomes are known from exquisite articulated remains, with the exception of the jawless galeaspids that are known from thousands of specimens of hundreds of species, but these are almost exclusively known from cranial remains. The postcranial anatomy of galeaspids was effectively unknown until the recent description of *Tujiaaspis vividus* [10] from the Silurian Chongqing Lagerstätte [11]. This is unfortunate since galeaspids are among the closest relatives of jawed vertebrates and, as such, they constrain the nature of the ancestral jawed vertebrate. The postcranial anatomy of *T. vividus* was wholly unexpected, with three dorsal fins and an approximately symmetrical hypochordal tail fin. Most surprisingly, *T. vividus* possesses paired elongate rigid fin-like structures that extended from the back of the pharynx and converging with the caudal fin, challenging conventional wisdom on the evolutionary origin of paired fins [10].

However, the precise anatomy of caudal fin in *T. vividus* remains unresolved and it is unclear whether this singular example of galeaspid postcranial anatomy is generally representative of galeaspids or specific to *T. vividus*. Here, we describe a new exceptionally preserved genus and species of galeaspid, *Foxaspis novemura* gen. et sp. nov., from a newly discovered Konservat-Lagerstätte from the Early Devonian (Pragian age, ∼410 Ma) of Guangxi, South China (Supplementary Figs 1 and 2). *F. novemura* has a broad circular and dorso-ventrally compressed headshield with a slender trunk and a flaring fan-shaped tail quite unlike *T. vividus*. Thus, the discovery of *F. novemura* suggests that the postcranial anatomy of galeaspids may have been as diverse as their crania, presumably reflecting broad ecological diversity. We use these new data to incorporate galeaspids into evolutionary analyses of swimming speed in early vertebrates, from which they have been omitted previously for lack of knowledge of their postcranial anatomy. These new analyses confirm that there is no directional trend towards increased metabolic activity within the gnathostome lineage.

## RESULTS

### Systematic palaeontology

Class Galeaspida Tarlo, 1967

Order Polybranchiaspidiformes Liu, 1965

Family Duyunolepididae P’an and Wang, 1978

Genus *Foxaspis* gen. nov.

**Figure 1.**
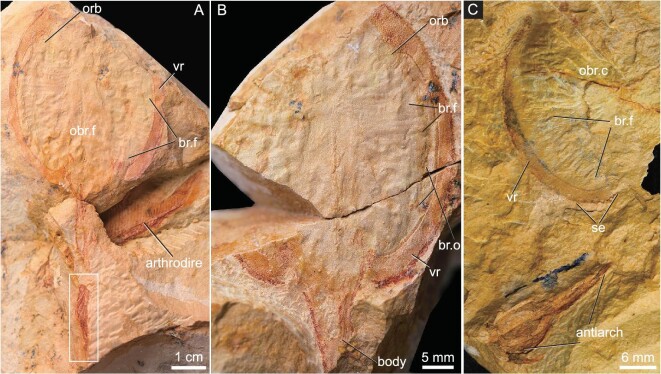
Photograph of *Foxaspis novemur* gen. et sp. nov. (A and B) A complete fish and its counterpart, the holotype, IVPP V30958.1a, b, associated with a complete arthrodiran fish, the outlined region in (a) is magnified in Fig. 3a. (C) An incomplete of headshield associated with a complete antiarch fish, paratype, V30958.3. Abbreviations: br.f, branchial fossa; br.o, branchial opening; obr.c, oralobranchial chamber; obr.f, oralobranchial fenestra; orb, orbital opening; se, serrated margin; vr, ventral rim.

**Figure 2.**
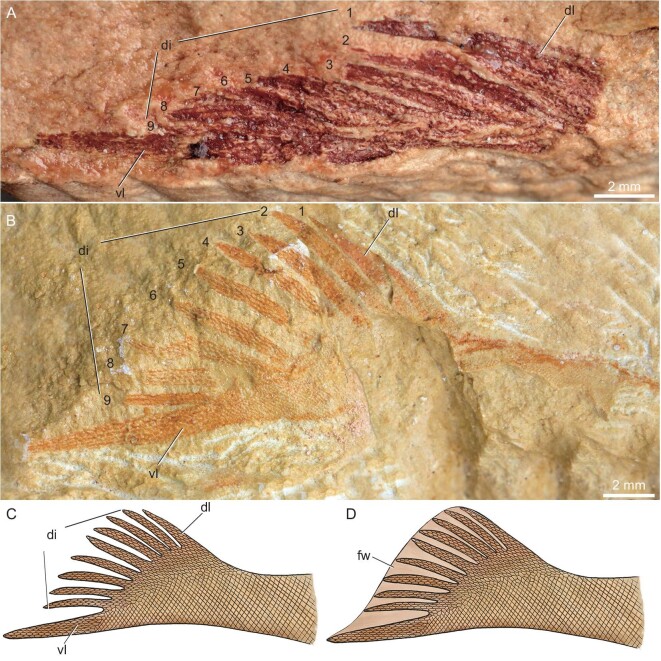
The tail of *Foxaspis novemur* gen. et sp. nov. (A) Magnification of the folded tail in the holotype, the region outlined in Fig. 1A; (B) an exceptionally preserved flared tail in the paratype, IVPP V30958.2a, b; (C) restoration of the tail, in lateral view; (D) restoration of the tail with fin web. Abbreviations: di, digitation; dl, dorsal lobe; fw, fin web; vl, ventral lobe.

**Figure 3.**
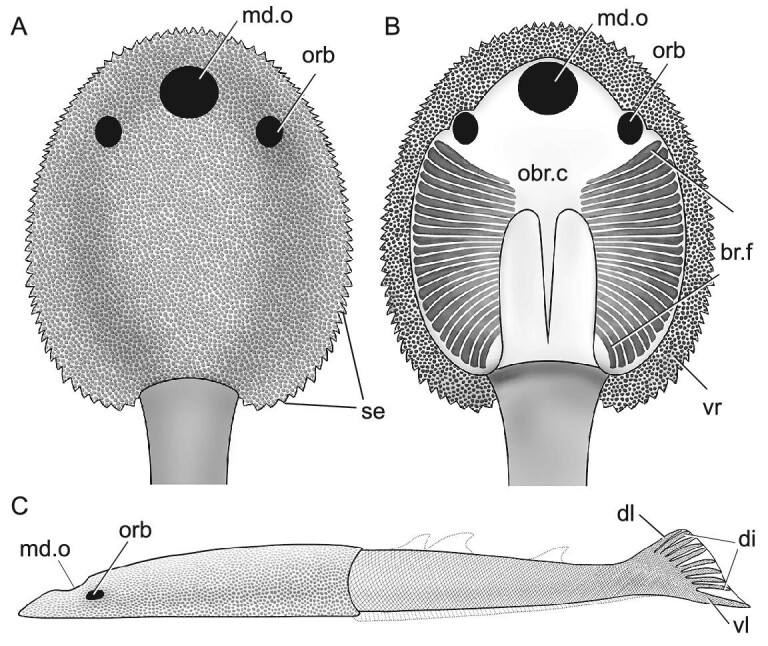
The restoration of *Foxaspis novemur* gen. et sp. nov. (A) In dorsal view; (B) in ventral view; (C) in lateral view. Abbreviations: md.o, median dorsal opening; others as in Figs 1 and 2.

*Foxaspis novemura* gen. et sp. nov.

(Figs 1–3, Supplementary Fig 3A, B and 4)

#### Etymology

After the nine-tailed fox, a creature spoken of in the ancient Chinese mythological bestiary, the *Shan-hai Ching* which is a compilation of mythic geography and myth. Latin *novem* meaning nine; Latin *-ura*, meaning tail.

#### Holotype

A complete headshield articulated with body and tail V30958.1a, b preserved together with a complete arthrodiran fish (Fig. 1A and B).

#### Paratype

An exceptionally preserved tail V30958.2a, b (Fig. 2B); an incomplete headshield V30958.3 is preserved together with a complete antiarch fish (Fig. 1C).

#### Locality and horizon

Tongmu Town, Jinxiu County, Laibin City, Guangxi Zhuang Autonomous Region, China, the Xiaoshan Formation, Pragian, Early Devonian (Supplementary Fig. 1).

#### Differential diagnosis

Medium-sized jawless fish up to 100 mm in length, which can be referred to Duyunolepididae of Polybranchiaspidiformes by its diagnostic oval-shaped headshield lacking cornual and inner cornual processes [12–15]. It differs from *Duyunolepis, Paraduyunaspis*, and *Lopadaspis* in its serrated margin of the headshield, differs from *Neoduyunaspis* in its much larger size and more lateral position of the orbital opening; ventral rim is thin, but nearly aequilate with a width of 4.0 mm, which is similar to that of *Lopadaspis*, but clearly differs from that of *Duyunolepis*; at least 25 pairs of branchial fossae [16]; ornamentation of the headshield composed of tiny granular tubercles; an elongate trunk with a length of 49.0 mm that comprised about half of its total length; the trunk is covered with minute square-shaped scales; the caudal fin is laterally compressed and obviously fork-shaped, which composed of a ventral chordal lobe and nine dorsal digitations; the caudal fin is clearly hypocercal in which the ventral chordal lobe is inclined downwards and much longer than the nine dorsal digitations; each digitation consists of lepidotrich-like rows of small elongate scales.

### Description

The holotype of *F. novemur* is about 100 mm in length, with a dorsoventrally flattened headshield and an elongated trunk (Fig. 1A and B). The headshield comprises about half of its total length with a length of 51.0 mm and a width of 42.0 mm. The headshield is oval in outline, but the cornual and inner cornual processes are both absent, which are regarded as morphological synapomorphies of Duyunolepididae of Polybranchiaspidiformes. The median dorsal opening is not preserved in the three specimens, but probably is oval-shaped as in *Duyunolepis, Paraduyunaspis*, and *Lopadaspis*. One orbital opening (orb, Fig. 1A and B) is preserved in the right part of the holotype. It is oval in outline and dorsally positioned, but very close to the lateral margin of the headshield. The long axis of the orbital opening is about 4.7 mm, and the short axis is about 3.4 mm. The ventral side of the headshield shows a large oval-shaped oralobranchial chamber (obr.c, Fig. 1A–C), which is encompassed by a pair of ventral rims and the dorsal part of the headshield. The ventral rim is thin, but nearly aequilate with a width of 4.0 mm, and a length of 56.0 mm (vr, Fig. 1A–C). At least 25 pairs of branchial fossae can be observed in the oralobranchial chamber of the holotype (br.f, Fig. 1A–C). Each branchial fossa opens to the exterior laterally by a round pore for the branchial opening (br.o, Fig. 1B), which aligned along the ventral rims. The diameter of each branchial opening is about 1.0 mm, whereas the length of the ventral rim posterior to orbital opening is about 35.6 mm. As such, the total number of branchial fossae in *F. novemur* is probably more than 30 pairs, which is close to that of *Lopadaspis* (32 pairs), and more than that of *Duyunolepis* (20 pairs) and *Paraduyunaspis* (24 pairs). The ornamentation of the headshield is composed of tiny granular tubercles, and there are about 12 tubercles per square millimetre.

The postcranial skeletal anatomy of *Foxaspis* is partly-preserved in the holotype (Figs 1A and 2A) and a flared tail is exceptionally preserved in the paratype V30958.2a, b (Fig. 2B). The trunk is elongate with a length of 49.0 mm and becomes increasingly laterally compressed in the caudal fin (Fig. 2A–D). The trunk inserts into the headshield just posterior to the branchial region, tapering to a fine point caudally (Figs 1A and 2). The trunk is covered with tiny rhombic scales arranged in oblique rows and there are about 20 scales per square millimetre (Figs 1A, B and 2). The caudal fin of *Foxaspis* is laterally compressed and clearly forked, which consists of a larger ventral lobe, a wider dorsal lobe and eight intermediate, narrower lobes (digitations) between them. The length of the ventral lobes both measure 11.3 mm in the holotype (Fig. 2A) and the paratype V30958 (Fig. 2B), whereas the length of the nine dorsal digitations from dorsal to ventral are measured in millimetres as follows: 7.1, 6.7, 6.6, 6.8, 6.8, ?, 6.7, 6.9, 6.8 in the holotype (Fig. 2A) and 6.7, 7.1, 7.0, 7.1, 7.1, 7.2, 7.3, 7.2, 6.9 in the paratype V30958 (Fig. 2B). Thus, the ventral lobe is not only longer but also thicker than the nine dorsal digitations and probably contains the notochord mass for the chordal lobe. The ventral chordal lobe is obviously inclined downwards indicating a hypocercal and asymmetrical condition. The fin web was supported by the ventral chordal lobe and the nine dorsal digitations, which directed towards the distal part of the web. The nine dorsal digitations aligned in an *en échelon* arrangement and had approximately the same length. Much smaller scales are observed at the dorsal and ventral margins of each digitation, presumably where the fin web attached to the lobe. Each lobe of the tail is linearly arranged by multiserial rows of lepidotrich-like scales per side (2–6 rows), which suggests that there were radial muscles allowing undulation of the fin web. Both the number of rows and the size of the scales are decreasing towards the posterior end of the fin. The scales on the tail are elongate, differing from the rhombic scales on the trunk. The scales on the intermediate digitations are smaller than the scales on the ventral lobes. In the holotype, all digitations and lobes are folded without gaps between them but, in contrast, they are fully flared in the paratype V30958 (Fig. 2B) with obvious gaps between the adjacent digitations. However, there are no scales between the gaps of the adjacent digitations (Fig. 2C), which may have been bridged by a soft fin web (Figs 2D and 3C).

### Estimation of swimming speeds in galeaspids and implications for the evolution of locomotion in vertebrates

Caudal fin morphology has widely been regarded as a key indicator of swimming capabilities and holds a high potential for predicting cruising and burst swimming speeds in aquatic vertebrates. A novel approach for predicting swimming speeds in extinct taxa was developed using phylogenetic generalized least squares (PGLS) linear models applied to caudal fin metrics in living fishes [17]. Their analysis of swimming speeds in stem-vertebrates was incomplete because the postcranial anatomy of galeaspids was unknown. This can now be remedied based on the discovery of the postcranial anatomy of *Tujiaaspis* and *Foxaspis*.

**Figure 4.**
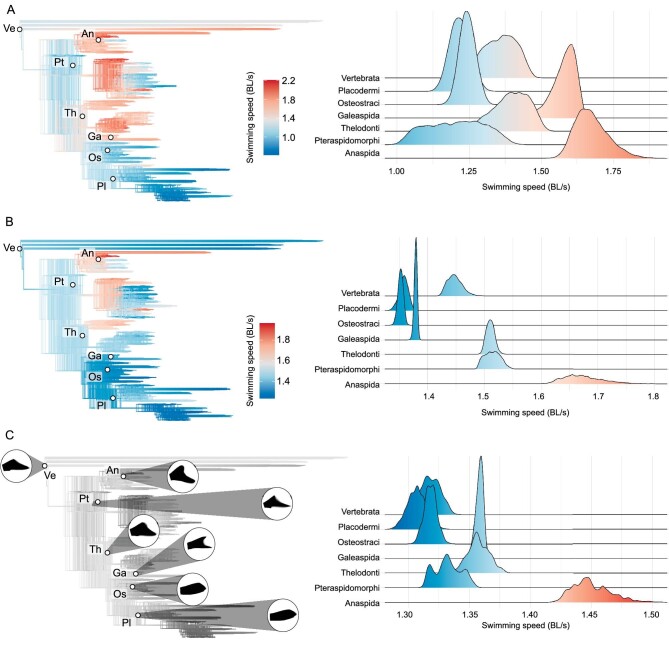
Ancestral cruising swimming speeds inferred for the main groups of Palaeozoic early vertebrates. Results derived from ancestral character state reconstruction of (A) non-sizenormalized speeds, (B) size-normalized speeds, and (C) caudal fin morphology. The results of each analysis are summarized as density trees with mapped ancestral speeds (in A and B) and caudal fin morphologies (in C); and density plots showing the ancestral speeds predicted for the main clades (left and right panels, respectively). A subsample of 100 trees randomly selected from the original pool are represented as density trees, while the whole pool of trees was considered for the density plots. Black caudal fin outlines represent the average of all the caudal fin morphologies inferred for each selected node in (C). Swimming speeds are in body lengths per second (BL/s). Taxa, Ve Vertebrata; An, Anaspida; Pt, Pteraspidomorphi; Th, Thelodonti; Ga, Galeaspida; Os, Osteostraci; Pl, Placodermi.

We estimated cruising swimming speeds based on a sample of 4500 phylogenies of stem- and crown-gnathostomes to account for temporal and phylogenetic uncertainty, considering alternative fossil calibrations and tree topologies (Fig. 4A). *Tujiaaspis* and *Foxaspis* were included as a clade, sister to Osteostraci plus Placodermi. We find an important variation among the swimming speeds predicted for the ancestral nodes of the main clades, with the highest values inferred for anaspids (mean ± SD equal to 1.675 ± 0.057 body lengths per second, BL/s) and galeaspids (mean ± SD equal to 1.580 ± 0.040 BL/s) and the lowest inferred for osteostracans, placoderms and pteraspidomorphs (mean ± SD equal to 1.244 ± 0.027 BL/s, 1.218 ± 0.034 BL/s and 1.203 ± 0.097 BL/s, respectively). Intermediate speed values are inferred for thelodonts (mean ± SD equal to 1.390 ± 0.067 BL/s) and the ancestral node of all vertebrates (mean ± SD equal to 1.357 ± 0.056 BL/s). Normalizing results by body length to account for differences in body size (i.e. considering the total body length of all taxa as 0.1 m) we recover a very similar pattern, with the only exceptions being the ancestral node of galeaspids and pteraspidomorphs which exhibit comparatively lower and higher speeds, respectively (mean ± SD equal to 1.675 ± 0.034, 1.514 ± 0.014, 1.510 ± 0.007, 1.448 ± 0.0123, 1.379 ± 0.002, 1.358 ± 0.008, and 1.352 ± 0.004 BL/s for anaspids, pteraspidomorphs, thelodonts, vertebrates, placoderms, and osteostracans, respectively) (Fig. 4B).

We further investigated the impact of inferred ancestral caudal fin morphologies on swimming speed ancestral estimates. For this, we predicted ancestral cruising swimming speeds of the main clades on reconstructions of their ancestral caudal fin morphology using geometric morphometrics and the established PGLS model. These analyses were performed in 1000 calibrated trees that were randomly sampled from the original pool (Fig. 4C). Patterns recovered by this alternative approach are comparable to those obtained from direct ancestral reconstruction of swimming speeds (see also Supplementary Fig. 5 for details on caudal fin morphospace occupation). Accordingly, the highest ancestral cruising swimming speed values are found in anaspids, followed by galeaspids, thelodonts, pteraspidomorphs, vertebrates, osteostracans, and placoderms (mean ± SD equal to 1.450 ± 0.016, 1.358 ± 0.003, 1.357 ± 0.009, 1.332 ± 0.011, 1.319 ± 0.007, 1.318 ± 0.005, and 1.307 ± 0.008, respectively).

**Figure 5.**
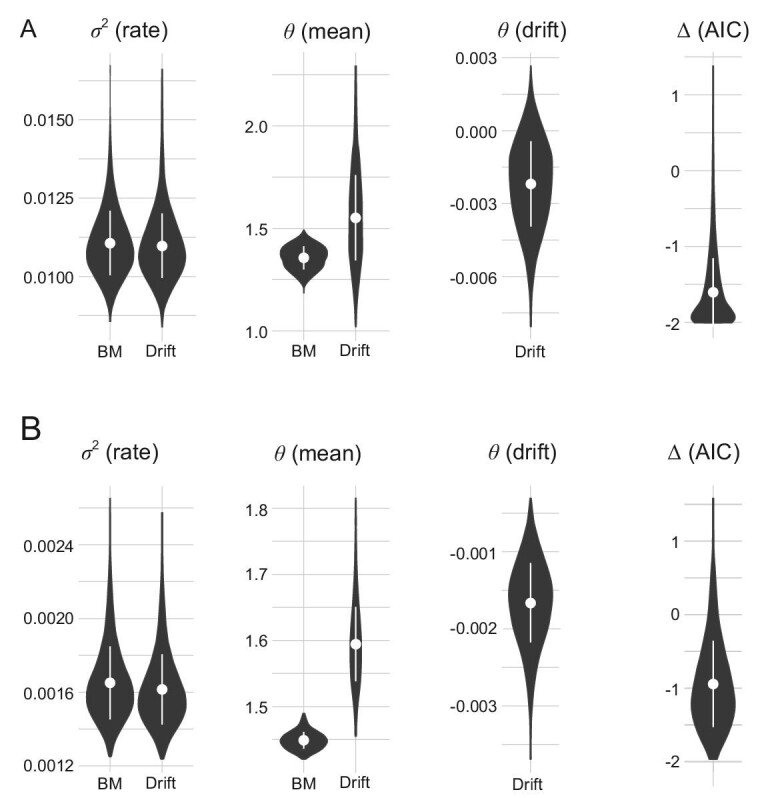
Evolutionary model fitting of cruising swimming speed in the main groups of Palaeozoic early vertebrates. Parameter estimates (σ^2^, rate of evolution; θ, trait mean; θ_drift_, drift of the trait mean) for Brownian motion and drift evolutionary models fitted to the datasets of (A) non–size-normalized speeds and (B) size normalized speeds, respectively. ΔAIC represents the difference between AIC_BM_ and AIC_Drift_.

To test for evolutionary trends in swimming speed capabilities we performed evolutionary model-fitting analyses. We explored the fit of two contrasting evolutionary models (a directional drift or ‘trend’ component versus a Brownian motion model) in the cruising swimming speeds estimated for stem-gnathostomes, using the pool of 4500 trees. When we consider non–size-normalized cruising swimming speeds (Fig. 5A), rate of evolution (σ^2^) parameter estimates notably overlap in both models (σ^2^_BM_ = 0.0111 ± 0.0010; σ^2^_Drift_ = 0.0110 ± 0.0010) while vaguely lower mean parameter values are predicted in the case of the Brownian motion model (θ_BM_ = 1.3577 ± 0.0559; θ_Drift_ = 1.5532 ± 0.2078). The drift parameter inferred for the drift model is close to zero but mostly negative (−0.0022 ± 0.0017). According to AIC scores, the Brownian motion model fits our data better than the drift model for all considered tree calibrations and topologies (AIC BM is generally smaller than the AIC Drift and, if larger, the difference is below two units; ΔAIC = −1.6022 ± 0.4511; Fig. 5A). When we consider size-normalized cruising swimming speeds, the pattern remains very similar, with a more evident difference on the mean parameter values predicted for each model (σ^2^_BM_ = 0.0016 ± 0.0002; σ^2^_Drift_ = 0.0016 ± 0.0002, θ_BM_ = 1.4477 ± 0.0128; θ_Drift_ = 1.5921 ± 0.0569; θ _Drift_ = −0.0016 ± 0.0005; ΔAIC = −0.9646 ± 0.5883) (Fig. 5B).

## DISCUSSION AND CONCLUSIONS

*Foxaspis novemura* is only the second galeaspid known from fully articulated remains and yet, nevertheless, suggests that the postcranial anatomy of galeaspids might be as diverse as cranial anatomy and this likely maps to their ecological diversity [18]. It is unclear whether *Foxaspis* possessed dorsal fins or paired appendages, but its strongly asymmetric caudal fin is quite unlike the approximately symmetrical caudal fin of *Tujiaaspis vividus* (Supplementary Fig. 3), though they are both hypochordal, with the notochord extending into the lower lobe of the fin, in contrast to the hypercaudal fins of osteostracans and jawed vertebrates. The contrasting flared and unflared caudal fin conditions in *Foxaspis* indicate that the caudal fin could be folded or flared (Fig. 2), reflecting modifiable control surfaces to increase or decrease surface area of the fin while generating thrust against the water. Lacking movable paired fins, the locomotion of *Foxaspis* necessarily relied heavily on the movements of the caudal fin, which played a major role in both the generation of thrust and maneuvering during swimming.

**Figure 6.**
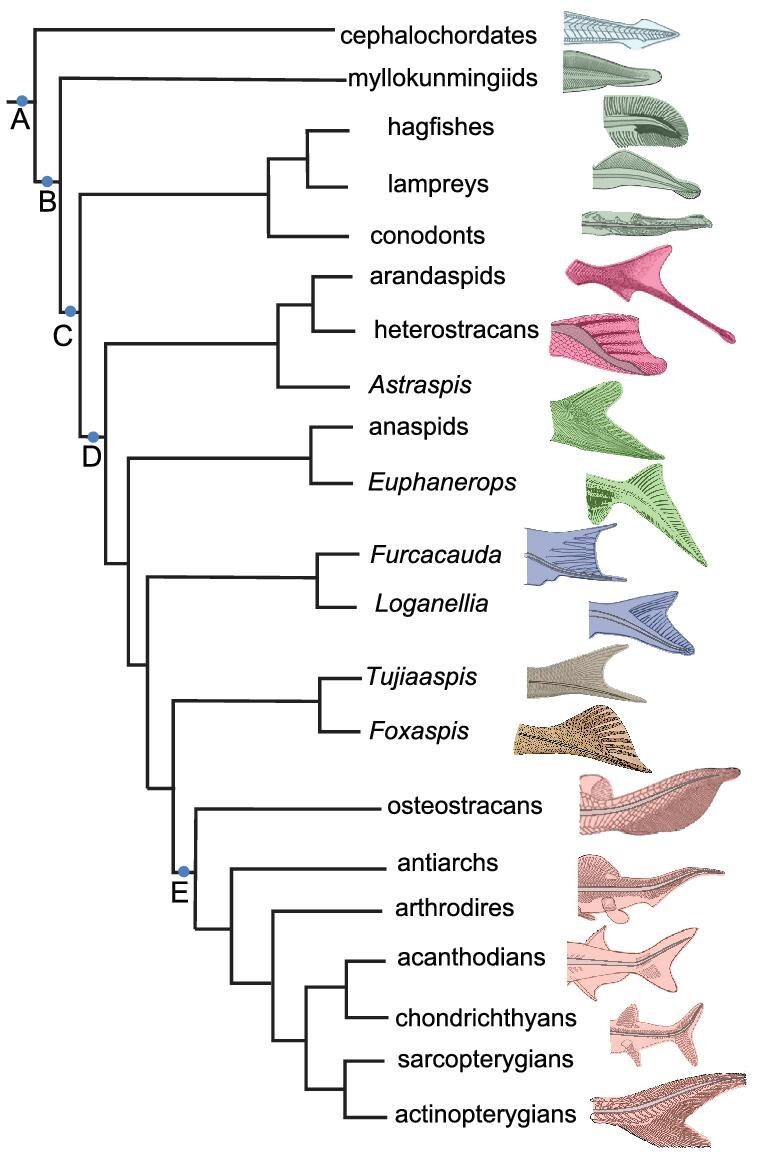
The evolution of caudal fins in early vertebrates (Tree topology after Ref. [4]). Node A, the origin of caudal fin; Node B, the origin of hypocercal tails and median fin fold; Node C, the origin of the individualized median fins; Node D, the origin of dermal fin rays probably with radial muscles; Node E, the origin of epicercal tail.

The caudal fin of *Tujiaaspis* is not completely preserved in any of the known specimens, but the preserved structure is suggestive of a forked tail with at least six, probably nine dorsal digitations as in *Foxaspis* [10] (Supplementary Fig. 3C and D). In both taxa, the digitations are covered with dermal scales organized in a lepidotrich-like linear arrangement. These similarities suggest that their shared ancestral species possessed a hypocercal tail with 6∼9 digitations covered with dermal fin rays, though it is not clear whether it exhibited a symmetrical or asymmetrical geometry. Nevertheless, the fork-tailed condition in *Foxaspis* and *Tujiaaspis* is most similar to heterostracans and furcacaudiform thelodonts in which the digitations vary from one to twelve (Fig. 6). Hypocercal tails occur in most living and fossil jawless vertebrates: *Haikouichthys* [19], cyclostomes (hagfishes + lampreys) [20–22], conodonts [23], anaspids [24], heterostracans [25], and thelodonts [26] (for a review, see [27]) (Fig. 6). The approximately symmetrical forked tail in galeaspid *Tujiaaspis*, heterostracans and the thelodont *Furcacauda* is probably a special condition of the hypocercal tail (Fig. 6), much like the teleost homocercal tail being a special condition of the epicercal tails of gnathostomes [27]. Cephalochordates have a simple isoceral caudal fin (Fig. 6), whereas osteostracans and gnathostomes are the only vertebrates possessing the unambiguously epicercal caudal fin [5,20] (Fig. 6 Node E). Therefore, a hypocercal tail seems to be primitive for vertebrates (Fig. 6 Node B), subsequently modified to an epicercal condition in the clade of osteostracans plus jawed vertebrates (Fig. 6 Node E), though this transition remains undocumented by transitional states [27]. The epicercal condition of osteostracans and jawed vertebrates represents a fundamental departure from the hypocercal tail fins of galeaspids and earlier branching ostracoderms. The innovation of an epicercal tail has been considered an adaptation to greater maneuverability, faster swimming speeds and a more active lifestyle. However, our analysis of swimming speeds among ostracoderms appears to belie this view.

Another of the most prominent characteristics of galeaspids is that the tail exhibits linearly arranged multiserial rows of lepidotrich-like scales per side (2–6 rows) as in the true fin rays of Osteichthyes. This suggests that there were radial muscles facilitating undulation of the fin web. The dermal fin rays were once regarded as a potential synapomorphy of anaspids plus gnathostomes [28], however, uniserial or multiserial dermal fin rays occur also in heterostracans, thelodonts and galeaspids indicative of a shared primitive condition (Fig. 6 Node D).

### Evolution of swimming speeds in association with the assembly of the gnathostome bodyplan

Caudal fin morphology is a key indicator of swimming capabilities and can be used to predict cruising and burst swimming speeds in aquatic vertebrates which, in turn, are good proxies for activity and metabolic level in living species [29–33]. This provides for an effective test of the ecological scenario underpinning the New Head hypothesis for early vertebrate evolution, which invokes a driven trend towards increasingly active food acquisition [34–36]. Ferrón and Donoghue used phylogenetic generalized linear models to estimate the swimming speeds of stem-gnathostomes based on their caudal fin shape [17]. However, their study entirely excluded galeaspids due to the absence of data on their postcranial anatomy. This is especially unfortunate given the proximity of galeaspids to the ancestor of jawed vertebrates, rendering galeaspids critical to inference on the nature of this ancestor. The limitation is remedied by the discovery of fully articulated remains of *Foxaspis* and *Tujiaaspis* (see Supplementary Table 1 for comparison of results derived from both studies).

Our analysis of swimming speeds in stem-gnathostomes indicates that galeaspids and anaspids have among the fastest cruising swimming speeds, with placoderms, osteostracans and heterostracans among the slowest (Fig. 4A). When these data are size-normalized (Fig. 4B), galeaspids are closer to this latter group, following thelodonts and pteraspidomorphs, with anaspids still the fastest. In these experiments, inferred swimming speeds were modelled over the tree, nevertheless, the ranking of cruising swimming speeds remains much the same when the evolution of caudal fin shape is instead modelled over the tree and cruising swimming speeds estimated *post hoc*, though galeaspids and thelodonts are then comparable (Fig. 4C).

Overall, cruising swimming estimates exhibit no phylogenetic structure since the fastest (anaspids) are among the most distant relatives of gnathostomes, while the closest relatives of the living jawed vertebrates (placoderms, osteostracans) are among the slowest. These results are perhaps surprising given the innovations in hyperchordal and hypercercal caudal fin morphology that osteostracans and placoderms exhibit relative to the plesiomorphic hypochordal caudal fin geometries of earlier branching plesia, including galeaspids and anaspids. Nevertheless, the absence of a trend is borne out by the evolutionary model-fitting analysis which rejected directional drift in favour of the Brownian motion model (Fig. 5). This is close as it may be possible to test the ecological scenario associated with the New Head hypothesis. Instead, our results imply that stem-gnathostomes were ecologically diverse, both within and among the component plesia of pteraspidomorphs, anaspids, thelodonts, galeaspids, osteostracans and the placoderm grades. This corroborates the analyses of cranial functional morphology [17,18,37], indicating that vertebrate ecological diversity was an intrinsic quality of early vertebrate communities and lineages, not predicated on the origin of the jaw or the diversification of crown-gnathostomes.

### Conclusions

We describe *Foxaspis novemura*, a new galeaspid genus and species, based on articulated remains from a newly discovered Konservat-Lagerstätte in the Early Devonian (Pragian age, ∼410 Ma) of Guangxi, South China. *F. novemura* is only the second galeaspid to be described with postcranial anatomy. It has a broad circular and strongly dorso-ventrally compressed headshield, with a slender trunk and a strongly asymmetrical hypochordal tail fin composed of ray-like digitations. This tail fin geometry contrasts the more approximately symmetrical caudal fin of *Tujiaaspis vividus*, the only other galeaspid known from complete postcranial remains, suggesting that, overall, the bodyform of galeaspids may be as diverse as their headshields. Our phylogenetically informed estimates of cruising swimming speeds indicate that galeaspids were more metabolically active than their more derived relatives, the osteostracans and placoderms. Overall, we reject the hypothesis that early vertebrate evolution is characterized by a directional trend towards increasingly active food acquisition, culminating in jawed vertebrates, as advocated by the New Head hypothesis. Stem-gnathostomes were ecologically diverse long before the origin of jawed vertebrates that doubtless diversified their feeding ecology within these settings.

## MATERIALS AND METHODS

### Specimens and provenance

All specimens of *Foxaspis* gen. nov. are permanently housed in the collections of the Institute of Vertebrate Paleontology and Paleoanthropology (IVPP), Chinese Academy of Sciences, Beijing and accessible for examination. The specimens were collected from the brown-yellow silty mudstone in the Xiaoshan Formation (Pragian, Early Devonian) near Tongmu Town, Laibin City, Guangxi Zhuang Autonomous Region, South China (Supplementary Fig. 1A). Being underlain unconformably by the Huangdongkou Formation of the Cambrian, the Early Devonian strata in this area are subdivided into Dayaoshan Group, Xiaoshan, Tonggeng, Luomai, Lutang, and Dale formations in ascending order [38–41] (Supplementary Fig. 1B). The fish-bearing Xiaoshan Formation, mainly dominated by brown-yellow argillaceous quartz siltstone intercalated with fine-grained argillaceous quartz sandstone, silty mudstone, and mudstone, reflecting a foreshore-shallow water marine environment [37,39]. The Xiaoshan Formation is conformably in contact with the underlying Dayaoshan Group and the overlying Tonggeng Formation [40,41]. The Konservat-Lagerstätte hosts diverse and disparate early fishes including several bizarre galeaspids, antiarchs, arthrodires and bony fishes (Supplementary Fig. 2A and B). The fish fossils are exceptionally well preserved with soft body and exquisite fin details (Supplementary Fig. 2A). In addition to fish fossils, plant remains such as *Zosterophyllum sinense* were collected from the same horizon and locality (Supplementary Fig. 2C and D), which indicates a mid-late Early Devonian (Pragian-Emsian) [42]. The age of the Xiaoshan Formation can also be determined as the Pragian base on the brachiopod *Orientospirifer wangi* which also has occurred at the Nakaolin (Nagaoling) Formation in Hengxian, Nanning, Guigang, and so on [40,43].

### Methods for swimming speed evolutionary patterns

Our analyses of swimming speed evolutionary patterns are based on the inclusion of the galeaspids *Tujiaaspis* and *Foxaspis* in the methodological framework developed in Ref. [17]. See the procedure in the supplementary data for further methodological details.

## DATA AND MATERIALS AVAILABILITY

All data are available in the manuscript or the supplementary materials. The Life Science Identifier (LSID) for the new genus and species has been deposited at ZooBank: Publication LSID: urn: lsid: zoobank.org: pub:88CC2848-34B6-47F7-A1A2-5B3B07E25E11; *Foxaspis* gen. nov. LSID: urn: lsid: zoobank.org: act:396BD648-C362-4112-A6F4-DE9CECF9CEBC; *Foxaspis novemura* gen. et sp. nov. LSID: urn: lsid: zoobank.org: act:5A44241D-A110-4D1E-BA1E-538A274733B0.

## Supplementary Material

nwad050_Supplemental_File
