# Supplementary material for: Postcranial disparity of galeaspids and the evolution of swimming speeds in stem-gnathostomes
Source: Natl Sci Rev. 2023 Feb 27;10(7):nwad050. doi: 10.1093/nsr/nwad050 (PMC10232041; doi:10.1093/nsr/nwad050)
Supplement: nwad050_Supplemental_File [file nwad050_supplemental_file.docx]

**Supplementary data**

**Supplementary Figures and Tab**

**Supplementary Figure 1.** Lithological column of *Foxaspis novemur* gen. et sp. nov. (A) and correlations of the Early Devonian fish-bearing strata in China and Vietnam. Abbreviations: Fm. Formation


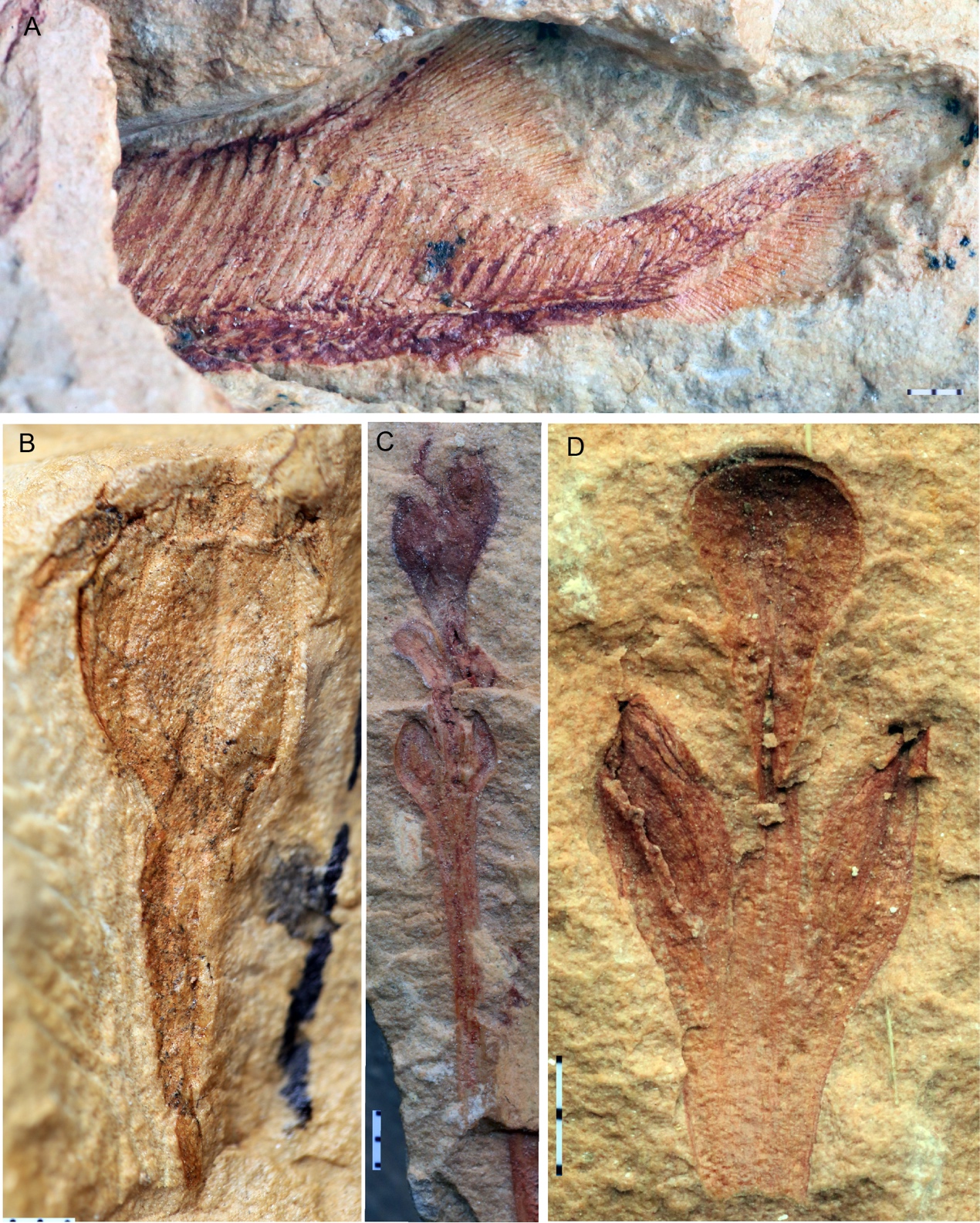


**Supplementary Figure 2**. The exceptionally preserved fishes and plants associated with *Foxaspis**.* A. a complete arthrodiran fish preserved together with the holotype of *Foxaspis* IVPP V30958.1; B. a complete antiarch fish preserved together with the paratype of *Foxaspis* IVPP V30958.3; C. D, the plant remains *Zosterophyllum sinense*


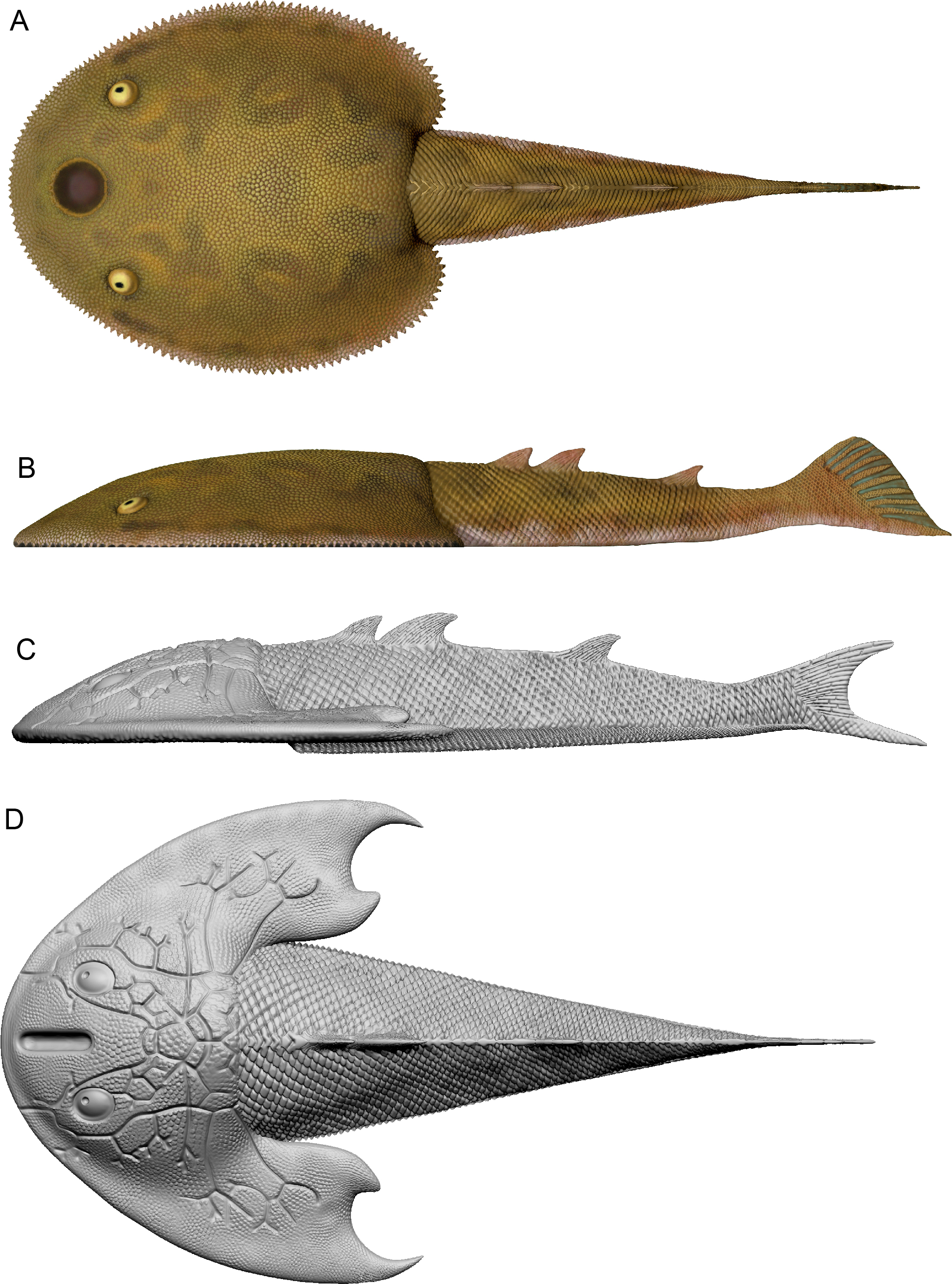


**Supplementary Figure 3.** The restoration and comparison of *Foxaspis* (A, B) and *Tujiaaspis* (C, D) (Credit Dinghua Yang)


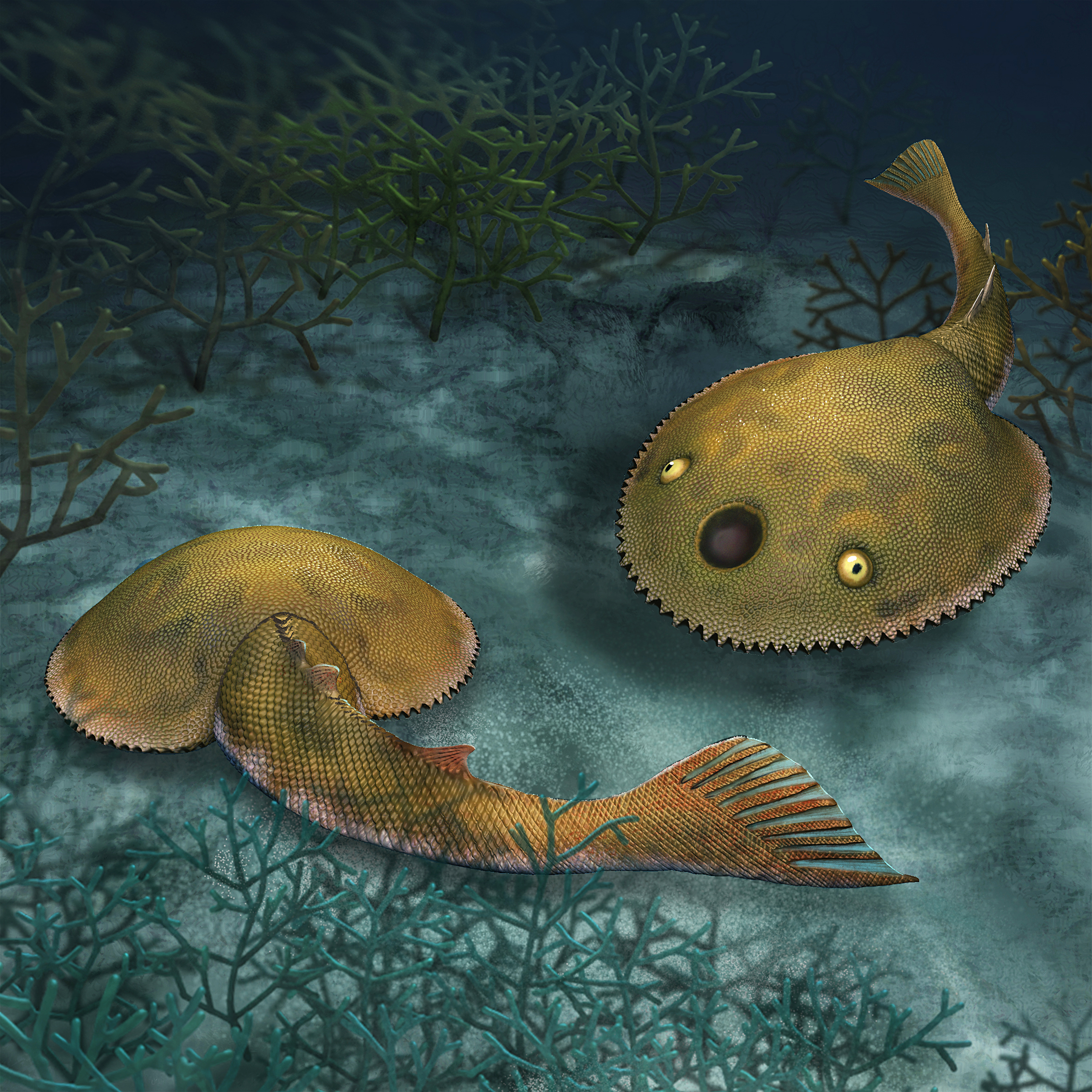


**Supplementary Figure 4**. The life restoration of *Foxaspis* (Credit Dinghua Yang)


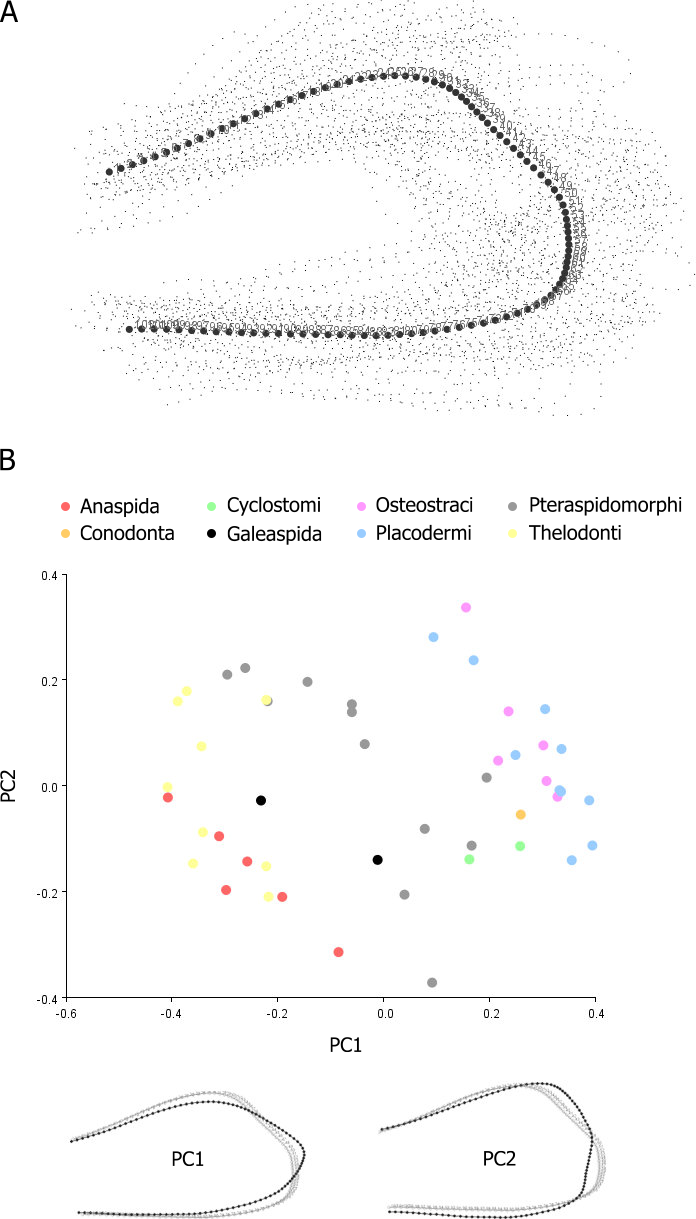


**Supplementary Figure 5.** Landmark configurations after Procrustes superimposition showing mean shape in bold (A) and PCA results derived from geometric morphometric analysis. Wireframes show shape changes towards positive scores of each PC axis (in black) compared to the mean shape (grey).

**Supplementary** **Table 1**. Comparison between ancestral swimming speeds inferred for the main clades of early vertebrates in this study and Ferron & Donoghue [1].

1. **Supplementary methods**

**Swimming speed evolutionary patterns**

Cruising swimming speeds of virtually all early vertebrates with known postcranial anatomy was predicted from a phylogenetically informed regression (PGLS), built with 161 living fish data that combined multiple predictors (i.e., total body length, caudal fin height to width ratio, swimming mode (cruising, burst) and swimming conditions (free, non-free swimming with specimens under controlled experimental conditions)). This PGLS model corresponds to the one with the best support, based on AIC criteria, from all the models checked in [1].

The sample of early vertebrates consisted of a total of 43 early vertebrates including Palaeozoic cyclostomes (Myxinidae and Petromyzontidae), jawless stem gnathostomes (Conodonta, Anaspida, Pteraspidomorphi, Thelodonti, the galeaspids *Tujiiaspis* and *Foxaspis*, and Osteostraci), and a representative sample of jawed stem gnathostomes (Placodermi).

We used a pool of 4500 phylogenetic supertrees including these taxa accounting for both phylogenetic and temporal uncertainty, also from [1]. *Tujiaaspis* and *Foxaspis* were included as a clade, sister to Osteostraci plus Placodermi and time calibrated using the R package ‘paleotree’[2].

We performed ancestral character state reconstruction analysis of predicted swimming speeds in the pool of 4500 phylogenetic trees using maximum likelihood method implemented in the R package ‘phytools’[3] and the same analysis was repeated with size-normalized cruising swimming speeds (i.e. considering all taxa having a total body length of 0.1 meters). Alternatively, we reconstructed ancestral caudal fin morphologies and derived cruising swimming speed predictions from them. For this, we employed geometric morphometric analysis on the caudal fin of the 43 early vertebrate taxa, considering a total of 102 landmarks, including two landmark type I in the caudal fin base dorsal and ventral margins and 100 landmark type III equally interpolated along the caudal fin outline. Landmark digitization of *Tujiiaspis* and *Foxaspis* was performed on lateral-view reconstructions using TpsDig v.2.26 [4]. The rest of landmark configurations were taken from [1]. We then implemented generalised Procrustes superimposition in landmark coordinates of all specimens using the R package ‘geomorph’ [5] to remove variation in rotational, scale and translational differences between specimens. Ancestral morphologies were reconstructed in a random sample the original pool of trees of 1000 trees, also using ‘geomorph’[5]. We measured caudal fin variables in the ancestral morphologies and their cruising swimming speeds were inferred from the PGLS model. Here, we considered all taxa having a total body length of 0.1 meters.

Results were visualized as density plots and density trees with mapped ancestral speeds using the R packages ‘ggplot2’[6], ‘ggridges’[7] and ‘ggtree’[8]. We also visualized the average of all the ancestral caudal fin morphologies inferred for each node by obtaining Z projections in ImageJ v 1.53b [9].

Finally, we fitted evolutionary models including a drift or ‘trend’ component to our datasets of both non-size-normalized and size-normalized cruising swimming in order to explore the presence of evolutionary trends in the swimming capabilities of early vertebrates. We also fitted a second model representing Brownian motion (i.e., where the trait evolves via a ‘random walk’) and AIC was employed to compare the goodness of fit for both models. The rate of evolution (σ^2^) and trait mean (θ) were estimated by finding the maximum-likelihood parameter values for each model. For the drift model, we also estimated the drift of the trait mean values. The fit of evolutionary models and parameter estimations were carried out in the original pool of 4500 phylogenetic trees using the R package ‘geiger’[10]. We represented the results as density plots with mean and standard deviation values using the R package ‘ggplot2’[6].

1. **Supplementary references**

1. Ferrón HG, Donoghue PCJ. Evolutionary analysis of swimming speed in early vertebrates challenges the 'New Head Hypothesis'. *Commun Biol*. 2022; **5**(1): 863.

2. Bapst DW. Paleotree: an R package for paleontological and phylogenetic analyses of evolution. *Methods Ecol Evol* 2012; **3**: 803–7.

3. Revell LJ. Phytools: an R package for phylogenetic comparative biology (and other things). *Methods Ecol Evol* 2012; **3**: 217–23.

4. Rohlf FJ. *TpsUtil, file utility program, release version 1.38* (Software and Manual)*.* Stony Brook: Department of Ecology and Evolution, State University of New York at Stony Brook; 2006.

5. Adams DC, Collyer M, Kaliontzopoulou A*, et al.* Geomorph: Software for geometric morphometric analyses. *R package version 3.0.6* (Software), 2019.

6. Wickham H. *Ggplot2: elegant graphics for data analysis*. New York: Springer, 2016.

7. Wilke CO. *Ggridges: Ridgeline plots in ’ggplot2*’. Austin, TX, US: Wilkelab, 2018.

8. Yu G, Smith DK and Zhu H*, et al.* Ggtree: an R package for visualization and annotation of phylogenetic trees with their covariates and other associated data. *Methods Ecol Evol* 2017; **8**: 28–36.

9. Schneider CA, Rasband WS and Eliceiri KW. NIH Image to ImageJ: 25 years of image analysis. *Nature methods*. 2012; **9**: 671–5.

10. Harmon LJ, Weir JT and Brock CD*, et al.* GEIGER: investigating evolutionary radiations. . *Bioinformatics* 2008; **24**: 129–31.
